# A Multi-Centric Study Assessing Safety and Efficacy of Everolimus in Adult Chinese Patients With Tuberous Sclerosis Complex Associated Renal Angiomyolipomas

**DOI:** 10.3389/fonc.2022.871723

**Published:** 2022-07-04

**Authors:** Wenda Wang, Gang Guo, Guohai Shi, Xin Wei, Zhiquan Hu, Hanzhong Li, Xu Zhang, Dingwei Ye, Yushi Zhang

**Affiliations:** ^1^ Department of Urology, Peking Union Medical College Hospital, Chinese Academy of Medical Sciences and Peking Union Medical College, Beijing, China; ^2^ Department of Urology, the Third Medical Centre, Chinese PLA General Hospital, Beijing, China; ^3^ Department of Urology, Fudan University Shanghai Cancer Center, Shanghai, China; ^4^ Department of Oncology, Shanghai Medical College, Shanghai, China; ^5^ Department of Urology, Institute of Urology, West China Hospital, Sichuan University, Chengdu, China; ^6^ Department of Urology, Tongji Hospital, Tongji Medical College, Huazhong University of Science and Technology, Wuhan, China; ^7^ Department of Urology, Chinese PLA General Hospital, Beijing, China

**Keywords:** tuberous sclerosis complex, angiomyolipoma, everolimus, efficacy and safety, gender

## Abstract

**Background:**

Everolimus has been approved in China for adult patients with TSC-AML (tuberous sclerosis associated renal angiomyolipomas) not requiring immediate surgery and has been previously shown to be an effective treatment option for TSC-AML in the Chinese population

**Methods:**

This is an open label, single arm, multi-center Phase IV post-approval commitment study to further assess the safety and efficacy of everolimus in patients with TSC-AML who do not require immediate surgical intervention. The primary outcome was to evaluate the safety of everolimus while the secondary outcome was to evaluate AML response.

**Results:**

Treatment with everolimus was associated with a clinically meaningful best overall AML response rate of 70% (95% CI: 53.5, 83.4). Of the 38 evaluable patients, 37 (97%) patients experienced a reduction in the sum of volumes of target angiomyolipoma lesions relative to baseline. At Week 12 (n=38), the median percentage change in sum of target AML volume was −56.60%, which further changed by -59.96% at Week 24 (n=38), and by −64.41% at Week 48 (n=22). Throughout the study, renal function remained relatively stable. Patients with TSC associated lymphangiomyomatosis (LAM) (N=13) demonstrated a lower than expected rate of decline in pulmonary function tests (PFTs). Everolimus was generally well tolerated with no significant safety findings in Chinese patients. Most of the adverse events were of grade 1-2, and manageable with appropriate dose adjustments and supportive therapies. There were no treatment discontinuation due to AE and no treatment death was reported.

**Conclusions:**

Based on the efficacy and safety data presented in this study, the overall clinical benefit/risk assessment further supports the use of everolimus as a viable treatment option for Chinese patients with TSC-AML.

## Background

Tuberous sclerosis complex (TSC) is a rare autosomal dominant genetic disorder caused by mutations in the *TSC1* or *TSC2* genes, which affect up to 1 million people worldwide, and is estimated to have an incidence of 1 in 6000 births (1). It is a debilitating disorder associated with the development of multiple benign tumors (hamartomas) which are highly vascularized, most commonly in the brain, kidney, lung and skin (2, 3). TSC most commonly present with neurological symptoms such as epilepsy, cognitive impairment, autism and other behavioral disorders. The most prevalent clinical manifestation of TSC is epilepsy which affects up to 90% of patients, followed by renal angiomyolipomas (AMLs) and pulmonary lymphangiomyomatosis (LAM) (1, 4). Interestingly, LAMs appear almost exclusively in females than males (5).

AMLs occur in up to 80% of patients with TSC, which is often detected in childhood and continues into adulthood (1, 4, 6). These slow-growing harmatomas composed of abnormal blood vessels, immature smooth muscle cells and fat cells and can lead to chronic kidney disease, renal failure or life-threatening hemorrhage as they enlarge (7, 8). Lesions which are more than 4cm have increased formation of aneurysms, and increased risk of spontaneous rupture (9). Due to encroachment of AML lesions on normal renal cells, approximately 1% of patients eventually progress to end stage renal failure and require dialysis (6).

The primary goal of managing TSC-AML is preservation of renal function and nephrectomy is generally avoided if possible due to high incidences of complications and increased risk of renal insufficiency, end stage renal failure and poor prognosis following chronic kidney disease. An mTOR inhibitor is currently recommended as the 1st line treatment for asymptomatic AMLs larger than 3cm in diameter (10). Selective embolization, kidney-sparing resections or ablative therapies are acceptable 2nd line treatment choice. In acute hemorrhage, embolization followed by corticosteroids is the current recommended therapy (10). It is important for closer collaboration between different stakeholders such as international and country-level TSC organizations, clinicians and researchers to ensure advances in TSC research to benefit patients with TSC globally (11).

Everolimus is a rapamycin derivative that inhibits the mTOR pathway by acting on the mechanistic target of rapamycin complex-1 (mTORC1). Mutations in the *TSC1* and *TSC2* gene result in the constitutive activation of mTOR which regulates cell grown, proliferation and angiogenesis (12). The EXIST-2 study showed that everolimus was safe and effective in the management of TSL-AML in Western patients who were not at risk of hemorrhage (13–15), while our previous study had also shown that everolimus is an effective treatment option for TSC-AML in the Chinese population (16). Everolimus has been approved in more than 90 countries for the treatment of TSC-AML. In 2016, everolimus was also approved in China for adult patients with TSC-AMLs not requiring immediate surgery.

The current multi-centric study is conducted to further assess the safety and efficacy of everolimus in adult Chinese patients with TSC-AMLs lesions based on Chinese current clinical practice as well as fulfilling a post approval commitment (PAC).

## Methods

### Study Design

This is an open label, single arm, multi-center Phase IV study for adult TSC-AML patients who are not requiring immediate surgical intervention. 40 patients were planned to receive everolimus for 48 weeks. Everolimus 10 mg per day was administered orally, with dose modifications allowed on the basis of safety findings. (Appendix 1).

The study protocol were reviewed and approved by the appropriate review board of each participating institution. The study was conducted in accordance with the International Council of Harmonisation of Technical Requirements for Pharmaceuticals for Human Use (ICH)-Good Clinical Practice (GCP) guidelines and the ethical principles that are outlined in the Declaration of Helsinki 2008. Written informed consent was obtained from each patient before any study-specific procedures was performed. This study is registered on Clinicaltrials.gov (NCT03525834).

### Patients

The study population included Chinese male or female adult patients (≥18 years of age) who have been diagnosed with TSC-AML that do not require immediate surgical intervention. Key inclusion criteria included patients who are eligible for treatment with everolimus as per approved indication and presence of at least one AML ≥ 3 cm in its longest diameter using computed tomography (CT) or magnetic resonance imaging (MRI). The key exclusion criteria are listed below: 1) AML related bleeding or embolization during the 6 months prior to enrollment; 2) History of myocardial infarction, angina or stroke related to atherosclerosis; 3) Impaired lung function, defined as any of the following: For patients without LAM, known impaired lung function (e.g. FEV_1_ or DL_CO_ ≤ 70% of predicted); For patients with LAM, DL_CO_ ≤35%, or O_2_ saturations below 90% at rest, or O_2_ saturation ≤88% on 6 minute walking test with up to 6 L O_2_/minute nasal Oxygen; 4) Significant hematological or hepatic abnormality (e.g. hemoglobin ≤9g/dL, platelets < 100×10^9/L, or ANC < 1.5×10^9/L without supportive treatment of hematopoietic growth factor, transaminase levels > 2.5× the ULN, serum bilirubin > 2 × ULN); 5) Prior therapy with mTOR inhibitors (sirolimus, temsirolimus, everolimus); 6) Use of an investigational drug within the 30 days prior to enrollment.

### Outcomes and Assessments

The primary objective of the study was to evaluate the safety of everolimus in Chinese adults with TSC-AML not requiring immediate surgery. Secondary objective was to evaluate efficacy of everolimus by measuring AML response. Other secondary endpoints included AML progression and renal function change from baseline.

CT or MRI was used to evaluate AML lesions. Imaging assessments for response evaluation were performed at 12, 24, and 48 weeks after start of treatment and at end of treatment. The radiological imaging scans performed for this trial was submitted by the sites to undergo central radiological review. All measurable AML with longest diameter ≥ 1.0 cm was identified from each kidney, and up to five of the largest measurable lesions on each kidney seen at screening were identified as target AML. AML volume was defined as the sum of the volumes of the individual target AML. The individual target AML volume was calculated according to the formula: V= 4/3×π×D1/2×D2/2×D3/2 (where D1 and D2 were long and short diameters of maximum cross section, D3 was maximum supero-inferior diameter). AML response was defined as a reduction in AML volume of at least 50% relative to screening. In addition, AML response required satisfying all of the following criteria: 1) No new AML ≥ 1.0 cm in longest diameter were identified; 2) Neither kidney had shown increase in volume by more than 20% from nadir (where nadir is the lowest kidney volume obtained for the patient, separately for each kidney, previously in the trial including screening); 3) The patient did not have any AML-related bleeding of grade ≥2 (as defined by NCI CTCAE, version 4.03). AML progression was defined as one or more of the following: 1) An increase from nadir of 25% or more in AML volume to a value greater than screening; 2) The appearance of a new AML ≥ 1.0 cm in longest diameter; 3) An increase from nadir of 20% or more in the volume of either kidney to a value greater than screening; 4) AML-related bleeding grade ≥2 as defined by NCI CTCAE, version 4.03.

Safety data were collected by monitoring and recording all adverse events (AEs), including serious adverse events (SAEs) and AEs of special interest (AESI), regular monitoring of vital signs and physical conditions, laboratory assessments including hematology, chemistry, coagulation, urine analysis and pregnancy. AEs were assessed and graded according to the CTCAE version 4.03 and Grade 1 to 5 were used to characterize the severity of the AE. Pulmonary function tests (PFTs) were performed on all patients with LAM at baseline, week 12, 24 and 48. Renal function was assessed using the CKD-EPI formula at baseline and at subsequent time points in assessment schedule.

### Statistical Analysis

Descriptive statistics were used to summarize baseline data and safety or efficacy variables. Responses were summarized in terms of percentage rates with exact 95% confidence intervals. An exact binomial confidence interval (Clopper and Pearson 1934) (17) was used. No Kaplan-Meier curve is contained in the manuscript.

## Results

### Characteristics of the Patients

Between 9 November 2018 to 3 September 2019, 40 patients were enrolled from 5 centers to receive everolimus treatment. Their demographics and baseline characteristics are summarized in **Table 1**. The median age was 33.0 years (range: 18-60). Most of the patients, 37 (92.5%), presented with at least two major features of TSC. Thirty-four (85%) patients had >2 AML lesions at baseline. The major and minor diagnostic features are presented in **Table 2** below. Overall, 10 (25%) out of the 40 patients received prior therapy to treat medical conditions, majorly epilepsy (7.5%) and hypertension (7.5%). Thirty-five (87.5%) patients received concomitant therapy during the study.

**Table 1:** Demographics and baseline characteristics.

Demographic variable	Everolimus
	N=40
Age (years) (median, range)	33.0 (18-60)
Gender (n, %)	
Female	26 (65.0)
Male	14 (35.0)
Race (n, %)	
Asian	40 (100)
Weight (kg) (median, range)	62.0 (41.0-102.0)
Height (cm) (median, range)	163.5 (153.0-185.0)
Body mass index (kg/m^2^) (median, range)	23.61 (16.6-30.8)
WHO performance status n (%)	
0	33 (82.5)
1	6 (15.0)
2	1 (2.5)

**Table 2:** Patient and disease characteristics at baseline (N=40).

Characteristics variable	n (%)
**Major Features**	
AML (greater than or equal to 2)^a^	34 (85.0)
Angiofibromas (greater than or equal to 3) or fibrous cephalic plaque	28 (70.0)
Hypomelanotic macules (greater than or equal to 3, at least 5-mm diameter)	16 (40.0)
Ungual fibromas (greater than or equal to 2)	15 (37.5)
Lymphangioleiomyomatosis (LAM)^a^	13 (32.5)
Shagreen patch	11 (27.5)
Subependymal nodules	10 (25.0)
Subependymal giant cell astrocytoma^b^	3 (7.5)
Cortical dysplasias	1 (2.5)
**Minor Features**	
Multiple renal cysts	8 (20.0)
Nonrenal hamartomas	5 (12.5)
Confetti skin lesions	4 (10.0)

a A combination of the two major clinical features (LAM and AML) without other features does not meet criteria for a definite diagnosis.

b Includes tubers and cerebral white matter radial migration lines.

Majority of the patients (92.5%) had a range of 1-5 target AML lesions defined as being ≥1 cm in the longest diameter, with 16 patients (40%) having the largest AML lesion of between ≥4 cm and <8 cm. Nearly a third of patients (n=13, 32.5%) had AML lesions of ≥8cm (**Table 3**).

**Table 3:** Kidney CT/MRI assessment at baseline (N=40).

	n (%)
Longest diameter of the largest AML lesion	
≥ 8 cm	13 (32.5)
≥ 4 cm and < 8 cm	16 (40.0)
≥ 3 cm and < 4 cm	8 (20.0)
< 3 cm	1 (2.5)
Not evaluable*	2 (5.0)
Number of target AML lesions (≥ 1 cm in longest diameter)	
1-5	37 (92.5)
6-10	1 (2.5)
> 10	0
Not evaluable*	2 (5.0)
Sum of Volumes of target angiomyolipoma lesions (cm^3^)	
Median, range	116.0 (2.0-1744.0)

### Everolimus Exposure

The cutoff date of following up was September 25, 2020. The median duration of exposure to everolimus treatment was 48.21 weeks (range, 38.0-50.3). 32 (80.0%) patients were exposed at least 48 weeks. While only eight (20%) patients were exposed less than 48 weeks (range, 36-48). The majority of patients (67.5%, n=27) received the planned dose of 10mg everolimus per day. Thirteen (32.5%) patients had at least one dose reduction and 22 (55%) patients had at least one dose interruption with AEs being the main reason. One patient permanently discontinued everolimus due to progressive disease. The dose ajustments of the patients were shown in Appendix 2. The median Cumulative dose was 3257.5mg (range, 965-3520). The median dose intensity (Cumulative dose/Duration of exposure) was 9.74 mg/day (range, 2.9-10.0). The median relative dose intensity (Dose intensity/Planned dose intensity) was 97.45% (range, 28.7-100.0%). 67.5% patients received relative dose intensity of more than 90%.

### Treatment Efficacy

**Figure 1:**
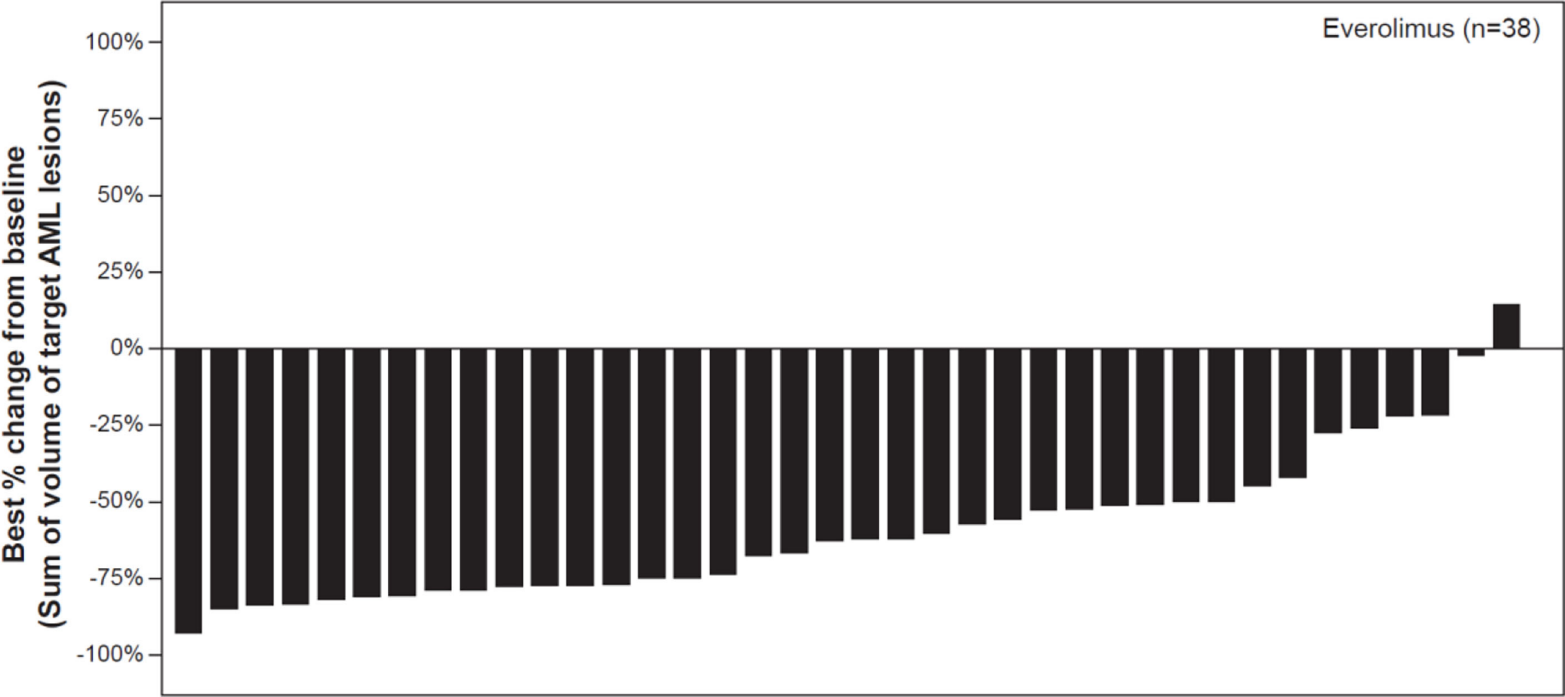
Waterfall plot of best percentage change from baseline in the sum of volumes of target angiomyolipoma lesions as per central radiology review. Decrease in best percentage change from baseline: 97.37%. Increase/zero change in percentrage change from baseline: 2.63%. *% change in sum of volumes of target AML lesions contradicted by overall AML response = PD: 0%. Patients for whom the best % change in sum of voumes of target AML lesions was not available and patients with overall AML response = Not evaluable were excluded from the graph, percentages above use n as denominator.

As per central radiology review, the best overall AML response rate was 70% (95% CI: 53.5, 83.4). Majority of patients had a clinical response (70%, 28/40 patients), or had stable disease (22.5%, 9/40 patients). One patient had disease progression while AML response was not evaluable in two patients due to unreadable tumor measurement. 97% of patients experienced a reduction in the sum of volumes of target AML lesions relative to baseline, however 1 patient had an increase from the sum of volumes of target AML lesions (**Figure 1**). 11 of 14 male patients (78.6% [95% CI 49.2 – 95.3]) had a clinical response, while 17 of 26 female patients (65.4% [95% CI 44.3 – 82.8]) had a clinical response.

**Figure 2:**
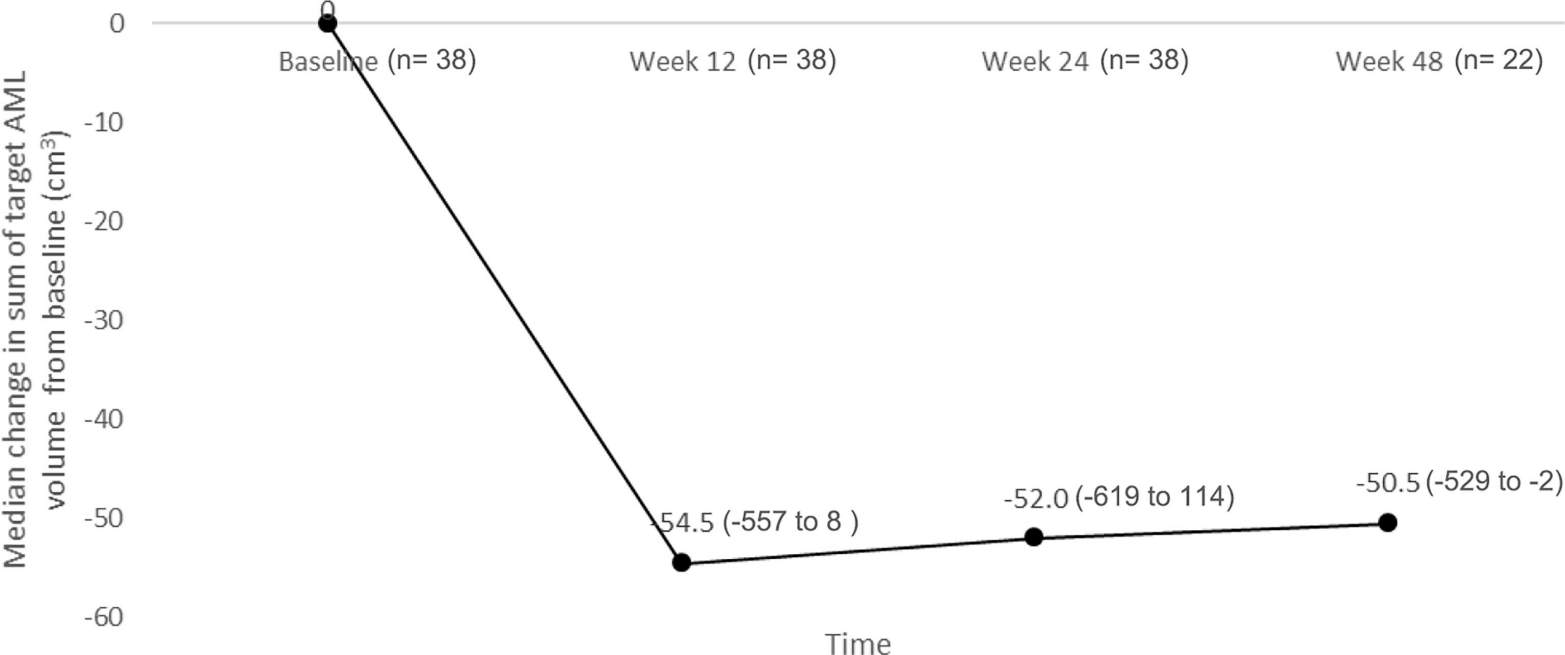
Median change in sum of target AML volume (median with range).

The reduction from baseline in sum of volumes of target AML lesions at week 12 persisted over time. At week 12, the median percentage change in sum of target AML volume was -56.60% (range, -90.6 to 19.5) (n= 38), which changed further to -59.96% (range, -92.7 to 38.3) at week 24 (n= 38), and -64.41% (range, -83.7 to -3.7) at week 48 (n= 22) (**Figure 2**).

Among the 28 patients with response, the median time to AML response was 2.8 months (95% CI: 2.8, 3.0). The estimated event free probability rates were 67.9%, 92.9% and 96.4% at Months 3, 6 and 12 respectively.

### Safety

All 40 patients in the study had at least one AE, of which 39 (97.5%) patients had treatment-related AEs. Fourteen (35%) patients had AEs of Grade ≥ 3 severity. Twenty (50%) patients had AEs that led to dose adjustment/interruption (All grade, 50%; Grade ≥ 3, 15%) and 38 (95%) patients experienced AEs which required additional therapy to resolve. The most frequent AEs that led to dose adjustment and/or interruption were stomatitis (8 patients, 20%) and mouth ulceration (4 patients; 10%). No deaths were reported in the study. The AEs and treatment related AEs which incidence greater than 10% are listed in **Tables 4**, **5**.

**Table 4:** Most frequent adverse events (incidences > 10%) by preferred term.

	Everolimus (N=40)	
	All Grades n (%)	Grade≥3 n (%)
Stomatitis	18 (45.0)	2 (5.0)
Mouth ulceration	15 (37.5)	1 (2.5)
Blood triglycerides increased	11 (27.5)	1 (2.5)
Blood cholesterol increased	10 (25.0)	0
Hypercholesterolaemia	10 (25.0)	0
Hypertriglyceridaemia	10 (25.0)	2 (5.0)
Anaemia	8 (20.0)	1 (2.5)
Blood lactate dehydrogenase increased	8 (20.0)	0
Protein urine present	8 (20.0)	3 (7.5)
Alanine aminotransferase increased	7 (17.5)	0
Blood creatinine increased	6 (15.0)	0
Menstruation delayed	6 (15.0)	0
Pyrexia	6 (15.0)	2 (5.0)
Weight decreased	6 (15.0)	0
White blood cell count decreased	6 (15.0)	0
Aspartate aminotransferase increased	5 (12.5)	0
Dermatitis acneiform	5 (12.5)	0
Folliculitis	5 (12.5)	0
Menstrual disorder	5 (12.5)	0
Diarrhoea	4 (10.0)	0
Nasopharyngitis	4 (10.0)	0
Neutrophil count decreased	4 (10.0)	0
Pneumonia	4 (10.0)	0
Proteinuria	4 (10.0)	2 (5.0)
Upper respiratory tract infection	4 (10.0)	0
Urinary tract infection	4 (10.0)	0

- Numbers (n) represent counts of patients.

- A patient with multiple severity grades for an AE is only counted under the maximum grade. MedDRA version 23.0, CTCAE version 4.03.

**Table 5:** Adverse events with suspected study drug relationship (incidence > 10%) by preferred term.

	Everolimus	
	N=40	
	All Grades	Grade ≥3
Preferred term	n (%)	n (%)
Number of patients with at least one event	39 (97.5)	11 (27.5)
Stomatitis	17 (42.5)	2 (5.0)
Mouth ulceration	15 (37.5)	1 (2.5)
Blood triglycerides increased	11 (27.5)	1 (2.5)
Blood cholesterol increased	10 (25.0)	0
Hypercholesterolaemia	10 (25.0)	0
Hypertriglyceridaemia	10 (25.0)	2 (5.0)
Blood lactate dehydrogenase increased	8 (20.0)	0
Protein urine present	8 (20.0)	3 (7.5)
Alanine aminotransferase increased	7 (17.5)	0
Anaemia	6 (15.0)	1 (2.5)
Blood creatinine increased	6 (15.0)	0
Menstruation delayed	6 (15.0)	0
Weight decreased	6 (15.0)	0
White blood cell count decreased	6 (15.0)	0
Aspartate aminotransferase increased	5 (12.5)	0
Dermatitis acneiform	5 (12.5)	0
Folliculitis	5 (12.5)	0
Menstrual disorder	5 (12.5)	0
Neutrophil count decreased	4 (10.0)	0
Pneumonia	4 (10.0)	0
Proteinuria	4 (10.0)	2 (5.0)

-Numbers (n) represent counts of patients.

-A patient with multiple severity grades for an AE is only counted under the maximum grade.

MedDRA version 23.0, CTCAE version 4.03.

Overall, six (15%) patients had at least one SAE, namely gastroenteritis, hemorrhoids, pneumonia, stomatitis, transient psychosis and uterine leiomyoma in one (2.5%) patient each. Three patients (7.5%) had SAEs of grade 3 severity. No Grade 4 and Grade 5 SAEs were reported in the study. Among SAEs, pneumonia (2.5%) and stomatitis (2.5%) were suspected to be related to everolimus, which were subsequently resolved with dose interruption of everolimus and related treatment of the specific AE.

AESIs are group of events for which there is specific clinical interest as a result of signals observed during the conduct of clinical studies. Overall, 39 (80%) patients had at least one of AESIs in this study. The AESIs of grade ≥3 severity were increased creatinine/proteinuria/renal failure (5 patients; 12.5%), stomatitis (3 patients; 7.5%), dyslipidemia (3 patients; 7.5%), cytopenia (1 patient; 2.5%), and hypersensitivity (1 patient; 2.5%). The incidence of AESI is presented in **Table 6**.

**Table 6:** Adverse events of special interest in this study by grouping.

	Everolimus (N=40)	
Safety Topic	All gradesn (%)	Grade ≥3n (%)
Any adverse event of special interest	39 (97.5)	11 (27.5)
Stomatitis	32 (80.0)	3 (7.5)
Dyslipidemia	29 (72.5)	3 (7.5)
Severe infections	17 (42.5)	0
Cytopenia	14 (35.0)	1 (2.5)
Increased creatinine/proteinuria/renal failure	14 (35.0)	5 (12.5)
Hypersensitivity (anaphylactic reactions)	11 (27.5)	1 (2.5)
Female fertility (including secondary amennorhoea	9 (22.5)	0
Haemorrhages	6 (15.0)	0
Muscle wasting/Muscle loss	6 (15.0)	0
Non-infectious pneumonitis	2 (5.0)	0

-Numbers (n) represent counts of patients.

-A patient with multiple severity grades for an AE is only counted under the maximum grade. MedDRA version 23.0, CTCAE version 4.03.

The incidences of mouth ulceration, hypercholesterolaemia and hypertriglyceridaemia were 50%, 30.8% and 30.8% in female patients, while all were 14.3% in male patients. The rates of stomatitis, increased ALT, increased blood cholesterol and dermatitis acneiform were 57.1%, 42.9%, 35.7% and 28.6% in male patients, and 38.5%, 3.8%, 19.2% and 3.8% in female patients respectively.

### Change in Renal Function

**Figure 3:**
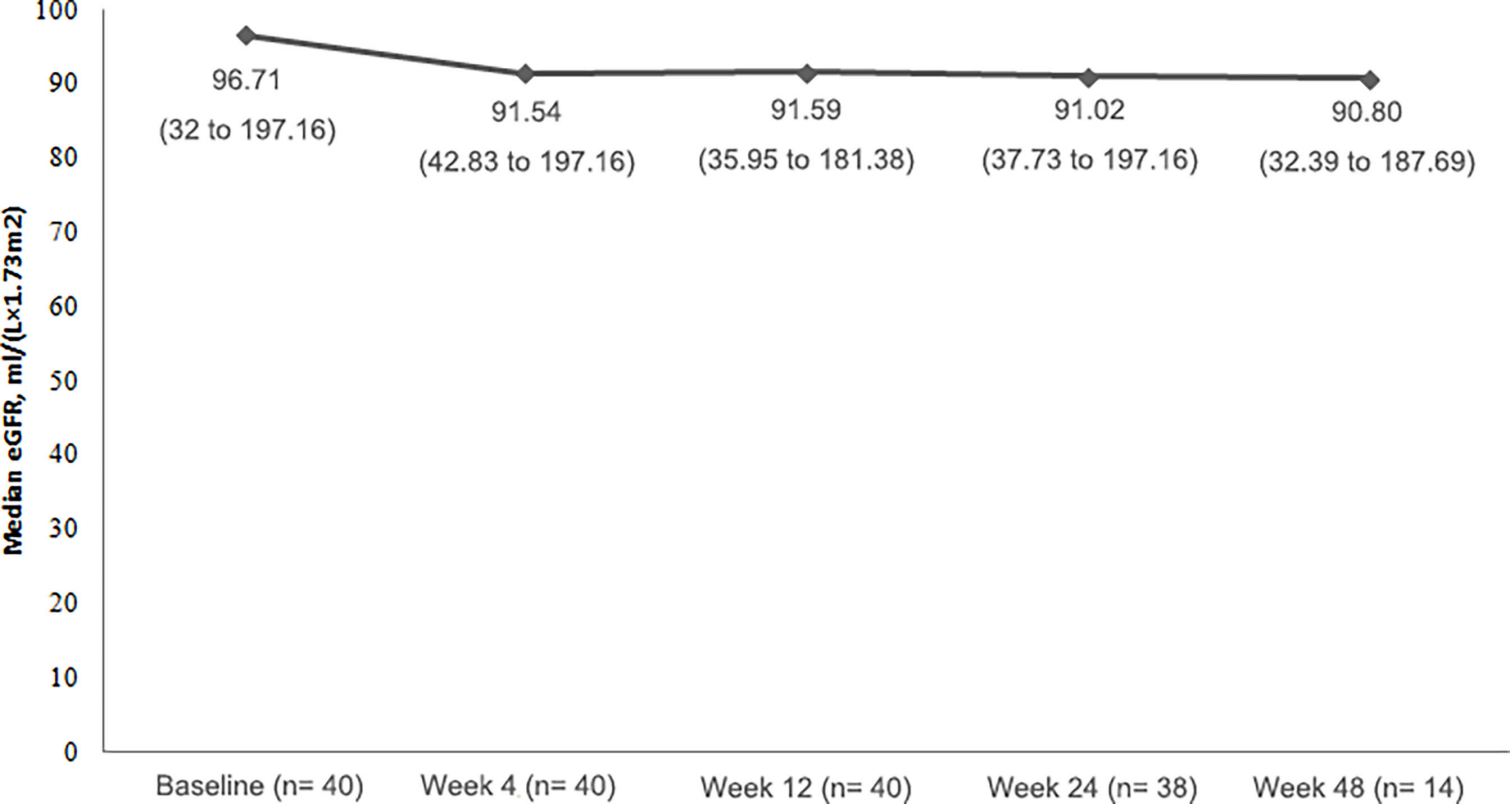
Median eGFR at each visit time point (median with range).

All of patients had eGFR ≥ 30mL/(min×1.73m^2^), and the majority of patietns had normal serum creatinine values (24 patients; 60.0%) at baseline. Throughout the study, median eGFR remained relatively stable (**Figure 3**).

Over the course of the study, although eGFR did not have obvious change, urine protein fluctuated and there was a noteworthy shift to a worse value: 23 (57.5%) patients had a negative urinalysis of urine protein at baseline, and only 3 (7.5%) patients remained negative during treatment. Four (10%) patients developed 3+ levels of protein in the urine at some point in the study.

### Change in Pulmonary Function

Among thirteen patients with LAM who were treated with everolimus, the changes from baseline in pulmonary function tests were assessed over the study. There were no significant deterioration in the pulmonary function throughout the study. The change from baseline over time were provided in **Table 7**.

**Table 7:** Change from baseline in pulmonary function tests by time point.

Value (Mean ± SD)	Baseline n=13	Week 12 n=13	Week 24 n=12	Week 48 n=4
FEV_1_, L	2.73 ± 0.34	2.77 ± 0.37	2.72 ± 0.42	2.58 ± 0.67
DL_CO_, %	88.73 ± 19.39	78.46 ± 15.60	81.10 ± 17.98	101.10 ± 24.18
FVC, L	3.18 ± 0.47	3.29 ± 0.53	3.26 ± 0.59	2.78 ± 0.95
FRC, L	2.99 ± 0.78	2.92 ± 0.56	2.87 ± 0.45	3.24 ± 0.81
Total lung capacity, L	5.11 ± 0.95	5.08 ± 0.53	4.99 ± 0.68	4.30 ± 1.17
Residual capacity, L	2.02 ± 0.61	1.93 ± 0.49	1.86 ± 0.57	1.93 ± 0.18

FEV_1_, expiratory volume in 1 second; DL_CO_, Diffusing capacity of the lung for carbon monoxide; FVC, Forced vital capacity, FRC, Functional residual capacity.

## Discussion

This was an open label, single arm, multi-center Phase IV study to assess the safety and efficacy of everolimus in the treatment of Chinese adults with TSC-AML not requiring immediate surgical intervention.

Everolimus was generally well tolerated with no significant safety findings in Chinese patients. Most of the AEs were of grade 1-2, and manageable with appropriate dose adjustments and supportive therapies. No new safety signals were reported in this study, and there were no treatment discontinuations due to AEs. It is important to note that pneumonia and stomatitis are known class effects of mTOR inhibitors. Good oral care and suitable oral care products should be recommended to these patients (19). Prophylactic steroid mouthwash has also been shown to reduce the incidences of everolimus-induced stomatitis (20) and could be considered for these patients, while patient education should be conducted on prevention and identification of infections. In addition to steroid mouthwash, other types of mouthwashes coupled with good oral hygiene have also been shown to be effective in controlling everolimus-induced stomatitis (21)

In the TOSCA study, AMLs were found to be significantly more prevalent in female patients, with higher numbers of AML lesions > 3cm, growing lesions as well as interventions for AMLs compared to the male population (22). However, the differences of treatment efficacy and AEs between gender were not reported previously. In our study, the response rate and the incidence of AEs seemed to be not the same by gender. However, due to the rather small numbers of patients in each gender group, the results should be interpreted with caution. More studies with larger number of patients should be conducted for more confirmatory evidence, which may help prevent AEs or better inform choice of treatment in regards to gender.

Positive efficacy results were comparable to those reported in previous studies with everolimus in TSC-AML (13–15). Best overall AML response rate by IRC was 70% (95% CI: 53.5, 83.4) which was further supported by magnitude and durability of the everolimus treatment effect in which a significant proportion of patients had evidence of sustained reduction in tumor volume at Week 48. This is similar to the result of our previous study in Chinese patients where the best overall AML response was 66.67% at 12 months of everolimus treatment (16). Almost all patients (97%) experienced a reduction in the sum of volumes of target AML lesions relative to baseline indicating a clinical benefit of everolimus for the treatment of patients with AML, where maximal benefit can be seen as soon as in 12 weeks. In our study, patients with LAM responded positively to everolimus, with less deterioration of lung function which was also shown in the EXIST-2 study (15, 23).

The clinical benefit of everolimus can be assessed not only by reducing tumor volumes, but also by a direct clinical benefit with regard to preservation of renal function. TSC patients usually have worse kidney function than the general population (24), however the deterioration in kidney function is not well understood. Patients with TSC on long-term everolimus have been shown to have stable renal function which concurred with our results here. The mechanism of everolimus inhibiting deterioration of renal function is not well known but it is hypothesized that mTOR activation may be the reason for decline of renal function in TSC patients, therefore early intervention with mTOR inhibitors may be beneficial in preserving renal function (25). Progression to stage 4 chronic kidney disease occurred only in patients who had compromised renal function at baseline (22) therefore close monitoring is recommended. Proteinuria is a known AE of mTOR inhibitor, and the majority of cases may be mild, transient or variable in patients with everolimus. Proteinuria > 1g/24h should be taken seriously and the dose of everolimus should be adjusted (25).

In this study, we have only followed patients on up to a year of everolimus treatment. Ideally, everolimus should be taken long term to maintain reduction in AML lesions. In the extension phase of EXIST-2, continued reduction in AML size was observed over a median of 28.9 months (14). However, there is a higher incidence of adverse events in Chinese patients on the standard recommended dosage of 10mg daily (16) which is also seen in our study. Long term use of everolimus is also associated with a higher risk of gonadal dysfunction and interstitial lung disease (26, 27). Several different method of everolimus administration have been studied which have reported similar magnitude of efficacy but with lower incidences of adverse events: intermittent administration, sequential administration and continuous low-dose administration (28–30). Additionally, clinicians may opt for surgical intervention or embolization to remove larger lesions which may lead to an early termination of everolimus treatment. More studies would be required to understand the optimal treatment of these patients to prevent disease rebound.

No patients were excluded from the analysis sets due to COVID-19 protocol deviation. The changes related to collection of safety assessments due to COVID-19 did not impact the data and safety analyses. However, 12 patients (30.0%) changed an assessment and 11 patients (27.5%) missed a visit due to COVID-19 pandemic. There were no major COVID-19 related protocol deviations reported resulting in any patient excluded from the analysis sets. As the impact was minimal, a sensitivity analysis was not performed.

In general, there are several limitations in our study. First, due to the small numbers of samples in our study, the results (especially those between genders) should be interpreted with caution. Our conclusions should be further verified in studies with larger sample size in the future. Second, the therapeutic and follow-up time in our study is just 1 year. The long-term results of Chinise patietns just as those in EXIST-2 are lack. The cohort studies with much longer research duration are needed in the future.

## Conclusions

Based on the efficacy and safety data presented in this study, everolimus has been shown to have a good clinical benefit/risk profile in patients with TSC-AML. Treatment with everolimus is associated with early and durable responses, whilst decreasing tumor burden. Known side effects of everolimus are mild and manageable. No new safety signals arose from this study. Therefore, everolimus is a viable treatment option for Chinese patients with TSC-AML who do not require immediate surgical intervention.

## Data Availability Statement

The original contributions presented in the study are included in the article/**Supplementary Material**. Further inquiries can be directed to the corresponding author.

## Ethics Statement

The studies involving human participants were reviewed and approved by the ethics committee of Peking Union Medical College Hospital, the ethics committee of Chinese PLA General Hospital, the ethics committee of Cancer Hospital Affiliated to Fudan University, the ethics committee of West China Hospital, the ethics committee of Tongji Hospital. The patients/participants provided their written informed consent to participate in this study.

## Author Contributions

Conceptualization: HL, YZ. Investigation: HL, XZ, DY, WX, and ZH. Project administration: WW, YZ, GG, GS, WX, and ZH. Writing-original draft: WW. Writing-review and editing: YZ, GG, GS, WX, ZH, HL, XZ, and DY. All authors contributed to the article and approved the submitted version.

## Funding

The study was supported by Novartis China.

## Conflict of Interest

HL, XZ, DY, WX, and ZH have served as investigators on this study and received research grants (to their institutions) from Novartis. This does not alter our adherence to the policies on sharing data and materials. Novartis was involved in the study design, collection, analysis, interpretation of data, and the decision to submit it for publication.

The remaining authors declare that the research was conducted in the absence of any commercial or financial relationships that could be constructed as a potential conflict of interest.

## Publisher’s Note

All claims expressed in this article are solely those of the authors and do not necessarily represent those of their affiliated organizations, or those of the publisher, the editors and the reviewers. Any product that may be evaluated in this article, or claim that may be made by its manufacturer, is not guaranteed or endorsed by the publisher.
