# Supplementary material for: A Multi-Centric Study Assessing Safety and Efficacy of Everolimus in Adult Chinese Patients With Tuberous Sclerosis Complex Associated Renal Angiomyolipomas
Source: Front Oncol. 2022 Jul 4;12:871723. doi: 10.3389/fonc.2022.871723 (PMC9290768; doi:10.3389/fonc.2022.871723)
Supplement: Supplementary file 1 [file Table_1.docx]

**Appendix 1-1. Details of study treatment schedule adjustments and dose levels**

|  | Starting dose  (level – 0) | Dose adjustments  (level – 1) | Dose adjustments  (level – 2) |
| --- | --- | --- | --- |
| Patients without hepatic impairment | 10 mg daily | 5 mg daily | 5 mg every  other day |
| Patients with mild hepatic impairment  (Child-Pugh grade A) | 7.5 mg daily | 5 mg daily | 5 mg every  other day |
| Patients with moderate hepatic  impairment (Child-Pugh grade B) | 5 mg daily | 5 mg every  other day | Not permitted |
| Notes:  ● Dose reduction should be based on the worst toxicity demonstrated after the most recent dose received.  ● Dose reduction below 5 mg every other day is not allowed. Treatment must be discontinued. | | | |

**Appendix 1-2. Dosing guidelines for study drug-related non-hematologic toxicities**

| Adverse drug reaction | Severity^1^ | Dose adjustment^2^ and management recommendations |
| --- | --- | --- |
| Non-infectious pneumonitis | Grade 1 Asymptomatic, clinical or diagnostic observations only; intervention not indicated | No dose adjustment required.  Initiate appropriate monitoring. |
|  | Grade 2 Symptomatic, medical intervention indicated; limiting instrumental ADL^3^ | Consider interruption of therapy, rule out infection and consider treatment with corticosteroids until symptoms improve to Grade ≤ 1. Re-initiate at a lower dose. Discontinue treatment if failure to recover within 4 weeks. |
|  | Grade 3 Severe symptoms; limiting self-care ADL^3^  O_2_ indicated | Interrupt until symptoms resolve to Grade ≤ 1. Rule out infection and consider treatment with corticosteroids. Consider re-initiating at a lower dose. If toxicity recurs at Grade 3, consider discontinuation. |
|  | Grade 4 Life-threatening respiratory compromise; urgent intervention indicated (e.g. tracheotomy or intubation) | Discontinue, rule out infection, and consider treatment with corticosteroids. |
| Stomatitis | Grade 1 Asymptomatic or mild symptoms; intervention not indicated | No dose adjustment required. Manage with non-alcoholic or salt water (0.9%) mouthwash several times a day. |
|  | Grade 2 Moderate pain; not interfering with oral intake; modified diet indicated | Temporary dose interruption until recovery to Grade ≤1. Re-initiate at the same dose. If stomatitis recurs at Grade 2, interrupt dose until recovery to Grade ≤1. Re-initiate at a lower dose. Manage with topical analgesic mouth treatments (e.g., benzocaine, butyl aminobenzoate, tetracaine hydrochloride, menthol or phenol) with or without topical corticosteroids (i.e. triamcinolone oral paste).^4^ |
|  | Grade 3 Severe pain; interfering with oral intake | Temporary dose interruption until recovery to Grade ≤1. Re-initiate at a lower dose. Manage with topical analgesic mouth treatments (e.g., benzocaine, butyl aminobenzoate, tetracaine hydrochloride, menthol or phenol) with or without topical corticosteroids (i.e. triamcinolone oral paste).^4^ |
|  | Grade 4 Life-threatening consequences; urgent intervention indicated | Discontinue and treat with appropriate medical therapy. |
| Other nonhematologic toxicities (excluding metabolic events) | Grade 1 | If toxicity is tolerable, no dose adjustment required. Initiate appropriate medical therapy and monitor. |
|  | Grade 2 | If toxicity is tolerable, no dose adjustment required. Initiate appropriate medical therapy and monitor. If toxicity becomes intolerable, temporary dose interruption until recovery to Grade ≤1. Re-initiate Afinitor® at the same dose. If toxicity recurs at Grade 2, interrupt until recovery to Grade ≤1. Re-initiate at a lower dose. |
|  | Grade 3 | Temporary dose interruption until recovery to Grade ≤1. Initiate appropriate medical therapy and monitor. Consider re-initiating at a lower dose. If toxicity recurs at Grade 3, consider discontinuation. |
|  | Grade 4 | Discontinue and treat with appropriate medical therapy. |
| Metabolic e vents(e.g. hyperglycemia, dyslipidemia) | Grade 1 | No dose adjustment required. Initiate appropriate medical therapy and monitor. |
|  | Grade 2 | No dose adjustment required. Manage with appropriate medical therapy and monitor. |
|  | Grade 3 | Temporary dose interruption. Re-initiate at a lower dose. Manage with appropriate medical therapy and monitor. |
|  | Grade 4 | Discontinue and treat with appropriate medical therapy. |
| ^1^ Severity Grade description: 1 = mild symptoms; 2 = moderate symptoms; 3 = severe symptoms; 4 = lifethreatening symptoms. ^2^ If dose reduction is required, the suggested dose is approximately 50% lower than the dose previously administered. ^3^ Activities of daily living (ADL) ^4^ Avoid using agents containing alcohol, hydrogen peroxide, iodine, and thyme derivatives in management of stomatitis as they may worsen mouth ulcers. | | |

**Appendix 1-3. Dosing guidelines for study drug-related hematologic toxicities**

| Adverse event | Severity^1^ | Dose Adjustment |
| --- | --- | --- |
| Thrombocytopenia (Platelet count decreased) | Grade 1 (<LLN-75.0x10^9^/L) | No dose adjustment required. |
|  | Grade 2 (<75.0-50.0x10^9^/L) | Temporary dose interruption until recovery to Grade ≤1. Re-initiate at the same dose. |
|  | Grade 3 (<50.0-25.0x10^9^/L) | Temporary dose interruption until recovery to Grade ≤1. Re-initiate at a lower dose. |
|  | Grade 4 (<25.0x10^9^/L) | Temporary dose interruption until recovery to Grade ≤1. Re-initiate at a lower dose. |
| Neutropenia (Neutrophil count decreased) | Grade 1 (<LLN-1.5x10^9^/L) | No dose adjustment required. |
|  | Grade 2 (<1.5-1.0x10^9^/L) | No dose adjustment required. |
|  | Grade 3 (<1.0-0.5x10^9^/L) | Temporary dose interruption until recovery to Grade ≤2 (ANC≥1.0x10^9^/l). Re-initiate at the same dose. |
|  | Grade 4 (<0.5x10^9^/L) | Temporary dose interruption until recovery to Grade ≤2 (ANC≥1.0x10^9^/l). Re-initiate at a lower dose. |
| Febrile neutropenia | Grade 3 (ANC<1.0x109/L with single temperature >38.3°C (101°F) or sustained temperature ≥38°C (100.4°F) for >1h) | Temporary dose interruption until recovery to Grade ≤2 (ANC≥1.25x10^9^/l) and no fever. Re-initiate at a lower dose. |
|  | Grade 4 (Life-threatening consequences; urgent intervention indicated) | Discontinue. |
| Any hematologic toxicity requiring study drug interruption for >28 days | | Discontinue. |
| ^1^ Grading based on National Cancer Institute (NCI) Common Terminology Criteria for Adverse Events (CTCAE) v4.03 | | |
