# Supplementary material for: A Multi-Centric Study Assessing Safety and Efficacy of Everolimus in Adult Chinese Patients With Tuberous Sclerosis Complex Associated Renal Angiomyolipomas
Source: Front Oncol. 2022 Jul 4;12:871723. doi: 10.3389/fonc.2022.871723 (PMC9290768; doi:10.3389/fonc.2022.871723)
Supplement: Supplementary file 2 [file Table_2.docx]

**Appendix 2. Dose adjustment and/or interrup during the study.**

| Subject | **Action taken** | **Reason** | **Start date (Study day)** | **End date (Study day)** | **Duration (days)** |
| --- | --- | --- | --- | --- | --- |
| 1000002 | Interruption | Stomatitis | 26 | 33 | 8 |
|  | Interruption | Stomatitis | 67 | 71 | 5 |
|  | Dose reduction (5mg qd) | Stomatitis | 72 | 85 | 14 |
|  | Interruption | Subject/guardian decision | 86 | 86 | 1 |
| 1000004 | Interruption | Malnutrition | 13 | 23 | 11 |
|  | Dose reduction (5mg qd) | Stomatitis | 24 | 26 | 3 |
| 1000005 | Interruption | Subject/guardian decision | -1^#^ | -1^#^ | 1 |
| 1000007 | Interruption | Stomatitis | 12 | 20 | 9 |
|  | Interruption | Stomatitis | 37 | 45 | 9 |
|  | Dose reduction (5mg qd) | Stomatitis | 46 | 338 | 293 |
| 1000009 | Interruption | Subject/guardian decision | 2019-03* | 2019-04* | - |
|  | Interruption | Subject/guardian decision | 64 | 64 | 1 |
| 1000010 | Interruption | Subject/guardian decision | 2019-04* | 2019-04* | - |
|  | Interruption | Subject/guardian decision | 2019-06* | 2019-06* | - |
| 1000013 | Interruption | Subject/guardian decision | -1^#^ | -1^#^ | 1 |
|  | Interruption | Subject/guardian decision | 7 | 7 | 1 |
|  | Interruption | Subject/guardian decision | 16 | 16 | 1 |
|  | Interruption | Subject/guardian decision | 2019-06* | 2019-06* | - |
|  | Interruption | Urinary tract infection | 233 | 251 | 19 |
| 1000014 | Interruptiom | Subject/guardian decision | 2019* | 2019* | - |
| 1000016 | Interruption | Subject/guardian decision | -1^#^ | -1^#^ | 1 |
|  | Interruption | Stomatitis | 12 | 19 | 8 |
|  | Interruption | Physician decision | 30 | 30 | 1 |
|  | Interruption | Blood triglycerides increased | 87 | 92 | 6 |
|  | Interruption | Blood triglycerides increased | 174 | 201 | 28 |
| 1000017 | Interruption | Subject/guardian decision | -1^#^ | -1^#^ | 1 |
|  | Interruption | Pyrexia | 5 | 11 | 7 |
|  | Interruption | Subject/guardian decision | 12 | 12 | 1 |
|  | Interruption | Stomatitis | 40 | 46 | 7 |
|  | Interruption | Subject/guardian decision | 71 | 75 | 5 |
|  | Dose reduction (5mg qd) | Stomatitis | 87 | 291 | 205 |
|  | Interruption | Mouth ulceration | 2020-03* | 2020-05* | - |
|  | Dose reduciton (5mg qod) | Physician decision | 292 | 336 | 45 |
| 1001003 | Interruption | Proteinuria | 166 | 172 | 7 |
|  | Interruption | Uterine leiomyoma | 209 | 217 | 9 |
|  | Interruption | Stomatitis | 248 | 254 | 7 |
| 1001007 | Interruption | Dosing error | 2019-07* | 2019-09* | - |
| 1003001 | Interruption | Stomatitis | 15 | 23 | 9 |
|  | Dose reduction (5mg qd) | Alanine aminotransferase | 173 | 226 | 54 |
| 1003003 | Interruption | Subject/guardian decision | 73 | 73 | 1 |
|  | Interruption | Subject/guardian decision | 2019-10* | 2020-04* | - |
| 1003005 | Interruption | Stomatitis | 51 | 60 | 10 |
|  | Interruption | Stomatitis | 91 | 107 | 17 |
|  | Interruption | Stomatitis | 135 | 139 | 5 |
|  | Interruption | Subject/guardian decision | 2020-01* | 2020-07* | - |
|  | Interruption | Stomatitis | 187 | 196 | 10 |
|  | Interruption | Stomatitis | 243 | 261 | 19 |
|  | Interruption | Stomatitis | 271 | 295 | 25 |
|  | Interruption | Stomatitis | 306 | 330 | 25 |
| 1003006 | Interruption | Subject/guardian decision | 20 | 21 | 2 |
|  | Dose change (20mg qd) | Subject/guardian decision | 2019-09* | 2019-11* | - |
|  | Interruption | Physician decision | 182 | 208 | 27 |
|  | Discontinuation | Progressive disease | 267 | - | - |
| 1005002 | Interruption | Rash | 101 | 105 | 5 |
|  | Interruption | Rash | 110 | 168 | 59 |
|  | Interruption | Subject/guardian decision | 2019-06* | 2019-11* | - |
|  | Dose reduction (5mg qd) | Rash | 169 | 336 | 168 |
| 1005003 | Dose reduction (5mg qd) | Hepatic failure | 32 | 42 | 11 |
|  | Interruption | Transient psychosis | 43 | 48 | 6 |
|  | Dose reduction (5mg qd) | Hypercholesterolaemia | 49 | 168 | 120 |
|  | Interruption | Subject/guardian decision | 169 | 336 | 168 |
| 1005006 | Interruption | Menstrual disorder | 146 | 152 | 7 |
| 1005008 | Interruption | Pneumonia | 93 | 120 | 28 |
|  | Dose reduction (5mg qd) | Pneumonia | 121 | 343 | 223 |
| 1005010 | Interruption | Pneumonia | 101 | 110 | 10 |
|  | Dose reduction (5mg qd) | Pneumonia | 113 | 336 | 224 |
| 1005011 | Interruption | Epistaxis | 120 | 126 | 7 |
| 1005012 | Dose reduction (5mg qd) | Folliculitis | 111 | 333 | 223 |
| 1005015 | Interruption | Mouth ulceration | 23 | 25 | 3 |
|  | Interruption | Mouth ulceration | 71 | 77 | 7 |
| 1005016 | Interruption | Mouth ulceration | 19 | 24 | 6 |
|  | Dose reduction (5mg qd) | Menstruation delayed | 36 | 336 | 301 |
| 1005017 | Dose reduction (5mg qd) | Mouth ulceration | 17 | 30 | 14 |
|  | Interruption | Anaemia | 31 | 38 | 8 |
|  | Interruption | Pyrexia | 108 | 111 | 4 |
| 1006002 | Interruption | Subject/guardian decision | 16 | 16 | 1 |
|  | Dose reduction (5mg qd) | Diarrhoea | 290 | 342 | 53 |

* The patients could not recall the exact start/end date.

^#^ The patients did not start treatment as planned.
